# Off-Flavours and Unpleasantness Are Cues for the Recognition and Valorization of Organic Wines by Experienced Tasters

**DOI:** 10.3390/foods9010105

**Published:** 2020-01-19

**Authors:** Mylena Romano, Mahesh Chandra, Mkrtich Harutunyan, Taciana Savian, Cristian Villegas, Valéria Minim, Manuel Malfeito-Ferreira

**Affiliations:** 1 Linking Landscape Environment Agriculture and Food (LEAF) Research Center, Instituto Superior de Agronomia, Universidade de Lisboa, Tapada da Ajuda, 1349-017 Lisboa, Portugal; mylena.romano@gmail.com (M.R.); mahesh.chandra@hotmail.com (M.C.); mkrtich.harutunyan@gmail.com (M.H.); 2 Department of Exact Sciences, College of Agriculture Luiz de Queiroz, University of São Paulo, 13418-900 São Paulo, Brazil; tvsavian@usp.br (T.S.); clobos@usp.br (C.V.); 3 Department of Food Technology, Federal University of Viçosa, Av. P.H. Rolfs, s/n, 36570-900 Viçosa, MG, Brazil; vprm@ufv.br

**Keywords:** organic wines, liking, willingness to pay, off-flavours, halo effect

## Abstract

The drivers of consumer acceptance concerning organic wines are not well understood. In particular, among wine professionals, there are anecdotal evidences claiming that consumers accept off-flavours that would not be tolerated if the wines were conventionally produced. Therefore, the aim of this study was to shed further light on this issue by tasting blind wines of both types of production using a tasting panel comprised by experienced individuals of several nationalities. The tasted wines were both conventional and organic and were with and without off-flavours. The same wines were evaluated in three tasting sessions where the given information was: (1) all wines were conventional, (2) all wines were organic, and (3) tasters were asked to guess the mode of production. A group of untrained tasters also rated the same organic wines in an informed session. The results showed that wines were significantly better scored and were given a higher willingness to pay value in the “organic” session. In addition, the experienced tasting panel produced a list of the most frequent sensory descriptors. When tasters were asked to guess the mode of production, wines that were supposed to be organic received a higher citation of off-flavours, such as “oxidized”, “reductive”, and “animal/undergrowth”. Moreover, an overall emotional response of unpleasantness was associated with the recognition of organic wines in the “guess” session. Untrained tasters rated the same organic wines with lower liking scores and were willing to pay less. In conclusion, off-flavours and their unpleasantness worked as a cue to identify wines supposed to be organic by experienced tasters. Their corresponding higher valorization could be explained by the psychological halo effect induced by the organic label. Contrarily, consumers did not show this halo effect, depreciating wines with unpleasant flavours irrespective of their mode of production.

## 1. Introduction

In recent years, consumers have become increasingly concerned about the effects of conventional agricultural production practices on both human and environmental health. Many agribusinesses have responded to these concerns, attracting customers by differentiating their products in crowded marketplaces [1,2]. The growing demand for environmentally sustainable products has created a boom in the field of green products mainly in developed countries [3,4]. Schäufele and Hamm [5] studied the consumers’ perceptions, preferences, and willingness to pay for wine with sustainability characteristics. The results suggested that producing and marketing wine with sustainability characteristics is a promising strategy for quality differentiation, particularly for wine that is both local and organic.

Among wine professionals, there is empirical evidence suggesting that the image of organic products has a stronger effect on consumer perception than the intrinsic characteristics [6]. The so-called halo or blurring effect can modify the sensory perception of products in terms of a positive image for organic and origin labelled food products and a negative one for more conventional products. In fact, Pagliarini et al. [7] found that consumers were not able to distinguish between organic and conventional wines in a blind tasting. Consumers preferred organic wine only in an informed situation. This indicates that the higher willingness to pay for organic wine may be due to consumers’ specific attitudes and involvement in sustainability issues. Furthermore, Wiedmann et al. [8] also showed that information about health, safety, and environmental issues increased consumers’ evaluation of organic wine. This halo effect received another piece of supporting evidence from the work of Apaolaza et al. [9] who found that when the wine was labeled organic, the same wine was perceived as having a finer, more intense, more floral and pleasurable fruity aroma, as well as a smoother texture and better body. This bias on hedonic evaluation and intention of purchase was totally mediated by increases in sensory ratings and perceived healthiness, providing a psychological or emotional explanation for this effect. Overall, these works [7,8,9] indicate that organic wines, in comparison with conventional ones, were associated with a higher quality and that a halo effect stemmed from storytelling, regardless of consumer knowledge and attitude towards organic products.

The research performed so far resulted from the responses of consumers with different wine knowledge and tasting wines without apparent off-flavours. As far as we are aware, there are no reports on the preference for organic wines with aromatic flaws using highly skilled wine tasters presumably more suited to detecting sensory differences between wines produced by both conventional and organic processes. Therefore, the aim of the present work was to evaluate the liking, willingness to pay and the sensory descriptors of organic wines using assessors with a high degree of wine knowledge and tasting training.

## 2. Materials and Methods

### 2.1. Tasting Panel Selection and Tasting Conditions

The tasters were students of the first and second year of the Vinifera EuroMaster of Viticulture and Oenology, held in Montpellier and in Lisbon, sharing a common descriptive language and knowledge of wines. A total of 48 students residing in Lisbon were recruited based on their interest and availability. Another panel was constituted by 47 untrained tasters recruited from faculty staff and other students. All subjects gave their informed consent for inclusion before they participated in the study.

The tastings took place in a sensory classroom of the Instituto Superior de Agronomia, in Lisbon, Portugal. The wines (30 mL) were poured into odour-free INAO-approved wine glasses with appropriate and constant temperatures (10 °C for whites and 18 °C for reds). The glasses were labelled with 3-digit codes and were covered by plastic Petri dishes, and the wine sequence was balanced among tasters. Once the first glass was tasted and the panelist moved to the second one and was not allowed to come back to the first glass. The white wines were tasted before the red wines. Between each sample, the panelists were instructed to eat unsalted crackers and cleanse their mouth with mineral water.

### 2.2. Tasting Sessions

The wines were tasted blind during three sessions in November and December 2018. The consumer session took place in February 2019. In the first session, tasters were informed that they were tasting “conventional wines” and in the second session the information changed to “organic wines”. In the third session, assessors were asked to guess the mode of production. Unexperienced tasters were informed about the organic nature of the wines during their session.

The wines are listed in Table 1 and were chosen according to their different sensory characteristics evaluated by 3 experts not participating in the study. Wines CB2016 and CT2015 represented organic wines with clean flavours, whereas the other 4 organic wines had different noticeable off-flavours. The conventional Burgundy wines (CC2013 and PN2015) were included as prototypes of classical European wines that are recognised as having initial off-flavours that disappear after leaving them to breathe [10]. Additional 4 conventional wines were included in the two first sessions to serve as distractors.

### 2.3. Tasting Questionnaire

The tasting sheet given to each individual included liking, familiarity scales, the willingness to pay (WTP) range, and a free sensory description list. The liking was determined using a Likert scale with the labels Dislike Very Much (1), Dislike (2), Neither Like nor Dislike (3), Like (4), and Like very Much (5). The participants were also asked to rate their familiarity from 1 (“I am not familiar with this wine”) to 5 (“I am familiar with this wine”). The WTP was calculated using the ranges 0–5, 5–10, 10–20, 20–30 €, >30 €, respectively. The average price was calculated by the sum of the product between the midpoint (arithmetic mean between the lower and upper bounds) of each class by the absolute frequency of the same class, divided by the sum of the frequencies.

Flavour descriptors were gathered according to similarity, and a frequency of citation was obtained following a free-choice profiling protocol [11], which assumed that panelists had previous common training [12].

### 2.4. Statistical Analysis

To compare the sensory acceptability, familiarity, and willingness to pay between the tasting sessions, a non-parametric pairwise Wilcoxon test was performed to determine whether two independent samples come from populations with the same distribution when the samples are independent and when measurements can be sorted on an ordinal scale [13]. The test was developed under the null hypothesis that the two samples have the same distribution at a significance level (α) of 5%, which is the probability of rejecting the null hypothesis when it is true. The forcefulness of the null hypothesis can be determined by calculating the p-value, which, when smaller than the chosen significance level, rejects the null hypothesis. The difference between the frequencies of descriptors’ citation was performed by a Pearson’s chi-squared test. The chi-squared statistic measures the difference between the actual counts and the expected counts (assuming validity of the null hypothesis that the categories are independent of one another in a significance level of 5%) [14]. All the statistical analysis was performed using R (https://www.r-project.org/).

## 3. Results

### 3.1. Tasting Panel Characterization

The trained tasting panel consisted of 48 participants, with ages ranging from 21- to 57-year-olds (average 27.5 ± 8.1 years old). The untrained tasting panel (47 participants) had a higher mean of age (38.2 ± 11.7 years old). The overall characterization of both panels is shown in Table 2.

### 3.2. Wine Liking

The average liking score on the “Conventional” session was 2.13, while the average on the “Organic” session was 3.76 (Table 3). When assessors had to guess the mode of production, the average score (2.56) was an intermediate between the two former tasting sessions.

The results show an increase of 76.5% in the appreciation when jurors were informed about the organic label, with individual wines augmenting from 45.8% to 113.9%. 

### 3.3. Wine Familiarity

The reported wine familiarity is shown in Table 4. Jurors revealed different familiarities according to the given session information. There was a tendency to increase from the first to the second session, revealing that the organic label induced an increase in familiarity. However, this difference was only observed for the wines CB2016, CR2015, and ST2012. The guess session yielded intermediate results.

### 3.4. Willingness to Pay (WTP)

The average WTP on the “Conventional” session was 4.44, which increased to 8.29 in the “Organic” session (Table 5). The “guess” session yielded intermediate values. All wines increased significantly from the first to the second tasting session (*p* < 0.05). In particular, the two Burgundy wines showed the highest relative increases in the “organic” session.

### 3.5. Flavour Descriptions

The large diversity of the individual descriptors that were generated was narrowed by gathering the free descriptors in families according to flavour similarity (Table 6) as widely described [15,16,17,18]. Interestingly, although not asked for, tasters also used a descriptor of an emotional nature related with the unpleasantness of the wines.

#### 3.5.1. Informed Tasting Sessions

The comparison between the description for each wine within the first and second wine tasting sessions was made with the whites separated from the from reds. The proportions of the citations of each descriptor cluster for the first two tasting sessions regarding all wines was analyzed by a chi-squared test with significance level of 5%.

Table 7 shows the citation frequencies for white wines. The organic wines CB2016 and VB2015 did not show differences between any descriptor family in both tasting sessions. CB2016 was mostly described with favourable flavors like fruity, floral, and fresh, corresponding to a clean wine sensory profile, which was to be expected based on the previous wine selection. On the contrary, VB2015 was a yellow, straw coloured wine dominated by an oxidized flavor clearly identified by the tasting panel.

The organic wine SB2016 elicited mostly animal/undergrowth and fruity flavours. However, the descriptor “Fruity” increased the frequency of citation from 45.8% to 77.1% in the organic session. Interestingly, the conventional Chablis wine (CC2013) showed a distinction between both tasting sessions in the descriptors “Fresh” and “Reductive”. When the information given was that all the wines tasted were conventional, 39.6% of the tasters mentioned “Fresh” and 56.2% mentioned “Reductive”, which increased to 83.3% and decreased to 22.9% in the “organic” session, respectively.

Overall, these results indicate that when wines were clearly clean (CB2016) or clearly faulty (VB2015), the description did not change according to the given information. When wines shared positive and negative flavours (SB2016 and CC2013), the organic label induced an increase the positive descriptors (“Fresh”) and/or a decrease in the negative descriptors as (“Reductive”).

In the red wines, the main descriptors obtained for the organic wines ST2012 and ST2014 showed that they were described similarly in both tasting sessions, being dominated by animal/undergrowth and fruity notes (Table 8). Regarding the organic CT2015, it was mostly described by “Fruity” and “Woody/Spicy” flavours, which is in accordance with the description of a clean wine. This wine increased the descriptors “Fresh”, “Fruity”, and “Floral” under the organic label. In addition, a tendency (*p* > 0.05) was observed in the animal/undergrowth descriptor with citations varying from 6.3 to 20.8%.

The conventional Pinot Noir (PN2015) was dominated by “Fruity” and “Animal/undergrowth”. The difference from ST2012 and ST2014 relied on the higher citation of “Fresh” in the Pinot Noir. For this wine, an increase in the “Floral” citation was observed from 0% to 16.7% in the organic session. Similar to CT2015, an increasing tendency was observed in the “Animal/undergrowth” citation varying from 58.3 to 70.8%.

Overall, as with the white wines, when the flavor profiles changed in the organic session, an increase in the positive descriptors was evidenced (“Fruity”, “Fresh”, and “Floral”). Interestingly, the descriptor “Animal/Undergrowth” showed a tendency in red wines to be more cited during the organic session.

#### 3.5.2. The “Guess” Session

**Figure 1:**
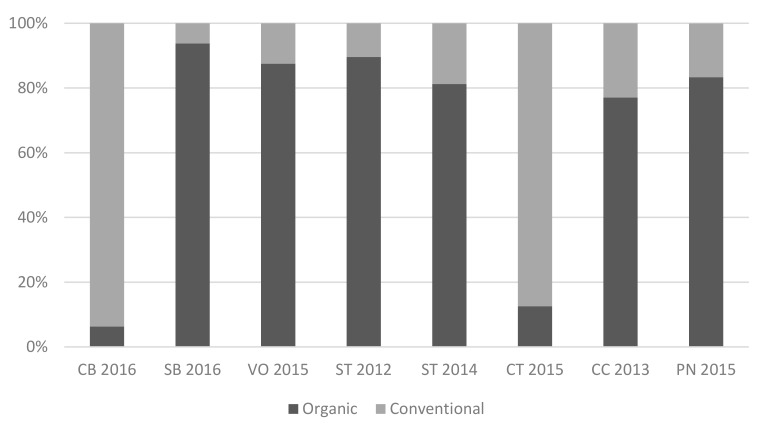
Mode of production chosen by the trained tasting panel for each wine.

In the “guess” session, panelists were asked to give their choice of the mode of production for the 8 tasted wines (Figure 1). Both the organic wines CB2016 and CT2015 were predominantly considered conventional by 93.8% and 87.5% of the tasters, respectively. Additionally, the two conventional wines from Burgundy, which were used as controls (CC2013 and PN2015), were mainly considered organic by 77.1% and 83.3% of the panelists, respectively. The other 4 organic wines (SB2016, VB2015, ST2012 and ST2014) were rightfully considered to be organic.

In order to understand which sensory features motivated the choice for the mode of production, the descriptors were merged according to the reported choice (Table 9). Among the 12 descriptors, 3 of them, as well as the emotional response, were shown to be different at *p* < 0.05. Regarding the descriptor “Animal/Undergrowth”, 51.0% of the tasters cited this descriptor when they thought the wine was organic and none used this term when the wine was considered to be conventional. The descriptor “Oxidised” also appeared more in the descriptions given by individuals who thought the wine was organic (36.1%) than of those who believed the wine was conventional (4.2%). Another descriptor following the same rationale was “Reductive”, being mentioned by 12.9% of the tasters when they thought it was organic and 0% of the testers when they thought it was conventional. Interestingly, in the Chablis wine, “Reductive” was less cited in the “organic” session compared to the “conventional” one (see Table 7), but when tasters had to guess, “Reductive” was only present in the wines thought to be organic. Moreover, for positive wine descriptors, such as “Fruity” and “Fresh”, there was a tendency (*p* > 0.05) to show higher percentages when wines were thought to be conventional.

#### 3.5.3. Overall Emotional Responses

In the sensory-free description, it was unexpected to find the citation “Unpleasant” given that it is not a sensory descriptor but an overall judgement of emotional nature. Table 10 shows its frequency of occurrence in the 3 tasting sessions. Interestingly, except for VB2015, with evident oxidised flavours, this emotional response was practically only used in the guess session. This response was mostly linked to wines thought to be organic. The lowest frequencies of the “Unpleasant” citation were observed in the organic wines CB2016 and CT2015, which were dominated by clean flavours.

**Figure 2:**
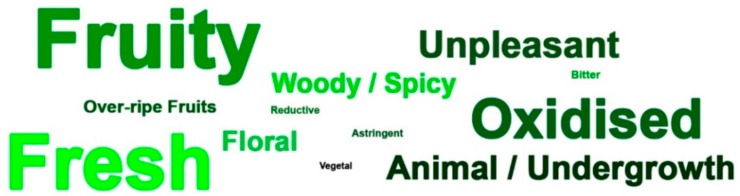
Word cloud of the descriptors elicited by the white wines considered to be organic (SB2016, VB2015, CC2013).

**Figure 3:**
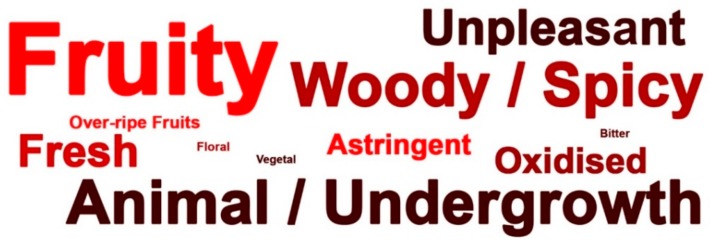
Word cloud of the descriptors elicited by the red wines considered to be organic (ST2012, ST2014, PN2015).

The responses given in the guess session may be considered as the psychological construct of a wine supposed to be organic wine by trained and knowledgeable tasters. Gathering the flavour descriptions and the emotional responses for white (Figure 2) and red wines (Figure 3) in a word cloud (font size according to frequency of occurrence) it becomes evident what may be regarded as the flavour and emotional profile expected to characterize organic wines for an experienced cohort.

### 3.6. Untrained Tasters

The liking, familiarity and willingness to pay (WTP) given by the untrained panel is listed in Table 11. Information about the organic mode of production was given to the tasters and so the results may be compared to the same wines tasted by the trained tasters in the “organic session”. Considering overall average scores, the unexperienced tasters either liked organic wines less or were willing to pay less for organic wines (*p* < 0.05) when compared to experienced tasters (see Table 3). The free descriptors generated revealed a wide heterogeneity of aromatic features and either pleasant or unpleasant response reactions, such as “nice”, “horrible”, or “weird”, frequently comparing the wines amongst themselves, consistent with the absence of previous training.

## 4. Discussion

A significant increase in sensory liking and the willingness to pay (WTP) was observed when trained tasters were informed about the organic mode of production. These results expose the influence of the organic “halo effect” on the perception of a wine, in accordance with Apaolaza et al. [9]. The novelty of the work here presented arises from the deliberate choice of tainted organic wines in order to assess the response of an educated cohort of tasters.

The results clearly show that tasters scored and valued wines much better under the “organic” label even when off-flavours were recognised. This heightened preference should be a result of the anticipated expectations since familiarity did not seem to explain this behavior. Ristic et al. [19] reported that freshness was associated to a positive sensory character while reduction was considered a negative aroma. Accordingly, in the present study, tasters changed the flavour description of conventional wines that were supposed to be organic or of flawless organic wines, increasing the citation of positive descriptors such as “fresh” or “fruity” and decreasing the citation of off-flavours like those associated with reduction. Thus, when tasters were informed about the mode of production, the positive perception of supposed organic wines was reflected in an increase of the perceived positive characters, as reported by Apaolaza et al. [9].

The most significant result was obtained with tainted organic wines and shows no significant difference in the sensory description between the conventional and organic sessions. For these wines, positive descriptors were not increased in the “organic” tasting and negative descriptors (such as “Animal/Undergrowth”, “Reductive”, “Oxidised”, and “Astringent”) were also not significantly decreased. It is likely that descriptors such as “Animal/Undergrowth” are not negative in the context of organic wines. Therefore, for equivalent sensory perception, a significant increase in liking and WTP was observed in organic wines. These outputs suggest that the acceptance of faulty wines was increased in the presence of an organic bias in the context of blind tasting. Moreover, it was not surprising that the classic European wines from Burgundy, used as prototypes for high reputation wines, usually not appreciated because of their initial off-flavours [10,20], were particularly liked in the “organic” session and not on the “conventional” one. With regards to the “Reductive” descriptor, in the Chablis wine, it was less cited in the “organic” session, but this descriptor was a cue for the “organic” guess. We hypothetise that this dual behaviour is a reflection of the emotional attachment that blurs flavour description. When there was no guess effort, more pleasant descriptors were used, and when a guess was required, the off-flavour cue was essential for the choice.

The importance of off-flavours in the organic wine recognition received another strong piece of evidence from the session where tasters had to guess the mode of production. Wines not presenting any sensory negative attributes were more likely to be identified as conventional by the tasters. The presence of off-flavours and their unpleasant character, most of which were mentioned in the guess session, were both cues to recognize organic wines. Therefore, the mode of production was not guessed but the presence of off-flavours was, which is to be expected from experienced individuals. Interestingly, the pleasantness evaluation was considered by Yeshurun and Sobel [21] to be an essential part of the sense of smell, being coined by these authors as an “emotional sense”. The organic label was worth more than the recognition of defects, as it bears a high affective load that overcomes the disgust caused of the flaws [22].

Spinelli et al. [23] evidenced the relations among sensory perception, liking, and emotions but the possibility of associating liking with unpleasant emotions was not observed. Therefore, it was striking that, in the case of organic wines, the psychological attachment was so strong that even a correctly recognized faulty wine could be more appreciated and deserved a higher price. This behavior may be explained under the frame of the top-down and bottom-up flavour brain processing mechanisms that are characteristic of experts and non-experts, respectively [24]. The previous knowledge about organic wine production induced a heightened pleasantness probably due to a neuronal process similar to that induced by other marketing actions [25]. On the contrary, non-experts using a bottom-up process did not show a comparable liking for faulty wines, even when informed about the production mode. Therefore, non-experts’ responses were more consistent with the wine’s intrinsic quality than those of experienced enology students that were more influenced by the “organic” extrinsic cues. These results were obtained with a low number of wines, but these wines were chosen to encompass a representative sample of the most common off-flavours found in Portuguese organic wines. Future studies should be performed with other off-flavours and with wines from other countries.

## 5. Conclusions

Overall, the results show that the “organic” concept is positively recognized by trained tasters, illustrating the well-known “halo effect” in sensory analysis, where, even before tasting, individuals are already prone to giving higher liking scores and willingness to pay ratings.

The novelty of this work stands in evidencing the acceptance of off-flavours when tasters were informed about the organic label of the wines. Surprisingly, off-flavours obtained a beneficial connotation as they were regarded as the cue for the recognition of organic wines. These results were obtained with a cohort of enology students that recognized off-flavours properly but did not penalize wines simply because they had the organic label. This fact poses a challenge to all involved in sensory analysis education because it is contrary to the pursuit of aromatic purity made by the precursors of wine science since the mid XX^th^ century, including Maynard Amerine in the University of California (Davis) or Émyle Peynaud and Jean Ribéreau-Gayon in Bordeaux University (France).

## Figures and Tables

**Table 1:** Wine description.

Wine Colour	Reference	Grape Variety	Vintage	Region ^a^	Production Method
White	CB2016	Síria	2016	DOC Beira Interior	Organic
	SB2016	Blend	2016	Portugal	Organic
	VB2015	Blend	2015	Portugal	Organic (not certified)
	CC2013	Chardonnay	2013	Chablis 1^er^ Cru	Conventional
Red	CT2015	Blend	2015	DOC Beira Interior	Organic
	ST2012	Blend	2012	Portugal	Organic
	ST2014	Blend	2014	Portugal	Organic
	PN2015	Pinot Noir	2015	AOC Bourgogne	Conventional

^a^ DOC: controlled designation of origin; AOC: appellation d’origine contrôlé.

**Table 2:** Tasting panel characterization.

Categories	Levels	Percentage (%)	
		Trained Tasters	Untrained Tasters
Gender	Female	50.0	46.8
	Male	50.0	53.2
Age	21–30	79.2	40.4
	31–40	12.5	17.0
	41–50	6.3	27.7
	51–69	2.1	14.9
Smoking habits	Non-smoker	91.7	89.4
	Smoker	8.3	10.6
Consumption frequency	Daily	14.6	10.6
	Two-three times per week	41.7	25.5
	One time per week	43.7	38.3
	1–2 times per month	0	25.5
	Occasionally (less than 1 time/month)	0	0
Self-reported knowledge	Beginner	27.1	55.3
	Intermediate	50.0	34.0
	Advanced	8.3	10.6
	Expert	14.6	0
Nationality	Portugal	56.3	55.3
	Germany	22.9	0
	Italy	16.7	4.3
	Brazil	12.5	36.2
	England	4.2	0
	France	2.1	0
	Colombia	2.1	0
	Hungary	2.1	0
	India	2.1	4.3

**Table 3:** Average sensory liking for each wine during the 3 tasting sessions.

Production Process	Wines	“Conventional” Session	“Organic” Session	Relative Increase Organic/Conventional (%)	“Guess” Session
Organic	CB2016	2.38	4.04	69.7	3.69
	SB2016	1.77	3.40	92.1	2.13
	VB2015	2.27	3.31	45.8	2.10
	CT2015	2.64	3.85	45.8	3.27
	ST2012	1.73	3.48	101.1	2.19
	ST2014	2.14	3.65	70.6	2.15
Conventional	CC2013	1.94	4.15	113.9	2.58
	PN2015	2.15	4.21	95.8	2.52
Average	-	2.13	3.76	76.5	2.56

**Table 4:** Average of familiarity for each wine during the 3 tasting sessions.

Production Process	Wines	“Conventional” Session	“Organic” Session	*p*-Value	“Guess” Session
Organic	CB2016	3.08	3.42	0.1	3.42
	SB2016	2.10	2.50	0.009	2.25
	VB2015	2.02	2.46	0.009	2.10
	CT2015	2.83	2.98	0.2	3.10
	ST2012	2.56	2.73	0.2	2.60
	ST2014	2.57	2.88	0.04	2.42
Conventional	CC2013	2.25	2.85	0.002	2.71
	PN2015	2.65	3.21	0.003	2.56

**Table 5:** Average willingness to pay (€) for each wine during the 3 tasting sessions.

Production Process	Wines	“Conventional” Session	“Organic” Session	Relative Increase Organic/Conventional (%)	“Guess” Session
Organic	CB2016	4.23	8.49	100.7	6.88
	SB2016	3.65	8.07	121.1	4.48
	VB2015	4.27	6.15	44.0	3.75
	CT2015	6.25	8.23	31.7	6.46
	ST2012	3.85	6.78	76.1	4.48
	ST2014	4.01	6.77	68.8	4.32
Conventional	CC2013	4.32	10.21	136.3	6.35
	PN2015	4.90	11.61	136.9	4.95
Average	-	4.44	8.29	86.7	5.20

**Table 6:** List of free sensory descriptors gathered in 12 overall families of flavor similarity.

Descriptor Family	Flavour Group	Free Descriptors
Fruity	Red fruits	Cherry, raspberry, redcurrant, strawberry, gooseberry
	Black fruits	Blackberry, blackcurrant, blueberry
	Citrus fruits	Grapefruit, lemon, lime, citrusy
	Tree fruits	Apple, pear, quince, apricot, peach
	Tropical fruits	Lychee, mango, pineapple
Over-ripe	Ripe/Jam fruits	Jam or compote of fruits
	Dried Fruits	Date, fig, almond, walnut, hazelnut, raisin
Floral	Floral	White flowers, violet, rose
Woody/Spicy	Spicy	Spicy
	Sweet spices	Black pepper, cinnamon, clove, curry, fennel, vanilla
	Woody	Oaky, fresh wood
	Roasted	Caramel, coffee, toasted, bread, smoky, chocolate
Vegetal	Dried Vegetal	Dried Vegetal
	Fresh Vegetal	Fresh Vegetal
	Liquorice	Liquorice
Animal/Undergrowth	Undergrowth	Earthy, humus, mould, mushroom, forest floor, wet leaves, truffle
	Animal	Brett, leather, musk, stable, mousy, barnyard, horse sweat
Oxidised	Oxidised	Apple cider, cooked apple, honey, acetaldehyde, volatile acidity
Fresh	Fresh	Good acidity
Bitter	Bitter	Bitter
Reductive	Reductive	Cooked cabbage
Astringent	Astringent	Astringent
Unpleasant	Unpleasant	Unpleasant

**Table 7:** Frequency of citation for each sensory descriptor elicited by white wines during “Conventional” and “Organic” tasting sessions *.

Descriptors	CB2016		SB2016		VO2015		CC2013	
	Conventional	Organic	Conventional	Organic	Conventional	Organic	Conventional	Organic
Fruity	87.5 ^a^	97.9 ^a^	45.8 ^a^	77.1 ^b^	33.3 ^a^	52.1 ^a^	70.8 ^a^	81.3 ^a^
Floral	52.1 ^a^	31.3 ^a^	27.1 ^a^	27.1 ^a^	2.1 ^a^	0.0 ^a^	10.4 ^a^	8.3 ^a^
Oxidised	18.7 ^a^	14.6 ^a^	20.8 ^a^	18.8 ^a^	87.5 ^a^	97.9 ^a^	4.2 ^a^	2.1 ^a^
Reductive	0.0 ^a^	0.0 ^a^	0.0 ^a^	6.3 ^a^	0.0 ^a^	0.0 ^a^	56.3 ^a^	22.9 ^b^
Animal/Undergrowth	0.0 ^a^	0.0 ^a^	56.3 ^a^	47.9 ^a^	2.1 ^a^	0.0 ^a^	25.0 ^a^	16.7 ^a^
Woody/Spicy	14.6 ^a^	12.5 ^a^	31.3 ^a^	16.7 ^a^	29.2 ^a^	33.3 ^a^	16.7 ^a^	31.3 ^a^
Over-ripe fruits	8.3 ^a^	0.0 ^a^	2.1 ^a^	0.0 ^a^	35.4 ^a^	35.4 ^a^	0.0 ^a^	2.1 ^a^
Vegetal	0.0 ^a^	0.0 ^a^	10.4 ^a^	22.9 ^a^	0.0 ^a^	2.1 ^a^	4.2 ^a^	2.1 ^a^
Fresh	66.7 ^a^	70.8 ^a^	29.2 ^a^	39.6 ^a^	10.4 ^a^	4.2 ^a^	39.6 ^a^	83.3 ^b^
Bitter	6.3 ^a^	4.2 ^a^	16.7 ^a^	8.3 ^a^	18.8 ^a^	6.3 ^a^	0.0 ^a^	0.0 ^a^
Astringent	2.1 ^a^	2.1 ^a^	0.0 ^a^	0.0 ^a^	4.2 ^a^	2.1 ^a^	0.0 ^a^	0.0 ^a^

* Numbers followed by the same letter in a row are not significantly different.

**Table 8:** Frequency of citations of each sensory descriptor elicited by red wines during “Conventional” and “Organic” tasting sessions *.

Descriptor	ST2012		ST2014		CT2015		PN2015	
	Conventional	Organic	Conventional	Organic	Conventional	Organic	Conventional	Organic
Fruity	72.9 ^a^	87.5 ^a^	93.8 ^a^	91.7 ^a^	77.1 ^a^	95.8 ^b^	95.8 ^a^	95.8 ^a^
Floral	0.0 ^a^	0.0 ^a^	14.6 ^a^	22.9 ^a^	8.3 ^a^	31.3 ^b^	0.0 ^a^	16.7 ^b^
Oxidised	8.3 ^a^	4.2 ^a^	4.2 ^a^	2.1 ^a^	0.0 ^a^	0.0 ^a^	0.0 ^a^	0.0 ^a^
Reductive	4.2 ^a^	4.2 ^a^	8.3 ^a^	0.0 ^a^	2.1 ^a^	2.1 ^a^	0.0 ^a^	14.6 ^a^
Animal/Undergrowth	89.6 ^a^	81.3 ^a^	64.6 ^a^	70.8 ^a^	6.3 ^a^	20.8 ^a^	58.3 ^a^	70.8 ^a^
Woody/Spicy	20.8 ^a^	35.4 ^a^	37.5 ^a^	39.6 ^a^	79.2 ^a^	77.1 ^a^	43.8 ^a^	47.9 ^a^
Over-ripe fruits	0.0 ^a^	18.8 ^a^	0.0 ^a^	10.4 ^a^	4.2 ^a^	4.2 ^a^	0.0 ^a^	0.0 ^a^
Vegetal	2.1 ^a^	2.1 ^a^	4.2 ^a^	2.1 ^a^	8.3 ^a^	14.6 ^a^	14.6 ^a^	10.4 ^a^
Fresh	0.0 ^a^	0.0 ^a^	4.2 ^a^	2.1 ^a^	0.0 ^a^	12.5 ^b^	29.2 ^a^	39.6 ^a^
Bitter	8.3 ^a^	6.3 ^a^	8.3 ^a^	2.1 ^a^	2.1 ^a^	4.2 ^a^	0.0 ^a^	0.0 ^a^
Astringent	20.8 ^a^	27.1 ^a^	14.6 ^a^	22.9 ^a^	39.6 ^a^	27.1 ^a^	29.2 ^a^	33.3 ^a^

* Numbers followed by the same letter in a row are not significantly different.

**Table 9:** Frequency of citations of sensory descriptors and emotional response elicited by wines considered to be conventional or organic.

Descriptors	Wines Considered as Conventional	Wines Considered as Organic	*p*-Value
Oxidised	4.1	36.1	5 × 10^−5^
Reductive	0.0	12.9	6 × 10^−6^
Animal/Undergrowth	0.0	51.0	<2 × 10^−16^
Unpleasant	3.1	51.4	2 × 10^−15^

**Table 10:** Percentage of citation of the emotional descriptor “Unpleasant” on each wine tasting session for each wine *.

Production Process	Wines	“Conventional” Session	“Organic” Session	“Guess” Session
Organic	CB2016	00.0 ^a^	00.0 ^a^	04.2 ^a^
	SB2016	00.0 ^b^	00.0 ^b^	33.3 ^a^
	VB2015	22.9 ^b^	10.4 ^b^	45.8 ^a^
	CT2015	00.0 ^a^	02.1 ^a^	02.1 ^a^
	ST2012	00.0 ^b^	00.0 ^b^	47.9 ^a^
	ST2014	00.0 ^b^	00.0 ^b^	39.6 ^a^
Conventional	CC2013	00.0 ^b^	02.1 ^b^	47.9 ^a^
	PN2015	00.0 ^b^	00.0 ^b^	47.9 ^a^

* Numbers followed by the same letter in a row are not significantly different.

**Table 11:** Preference and willingness to pay (WTP) of the organic wines by consumers in an informed session.

Wine Colour	Wine Code	Liking	Familiarity	WTP (€)
White	CB2016	2.89	2.36	5.0
	SB2016	2.96	2.72	6.1
	VB2015	2.51	2.17	6.4
Red	CT2015	2.89	2.53	7.1
	ST2012	2.83	2.66	5.5
	ST2014	2.64	2.45	5.9
Average	-	2.79	2.48	6.0
